# Potential of Pinewood Biochar as an Eco-Friendly Reducing Agent in Iron Ore Reduction

**DOI:** 10.1021/acsomega.3c09691

**Published:** 2024-03-15

**Authors:** Ajcharapa Chuanchai, Keng-Tung Wu

**Affiliations:** Department of Forestry, National Chung Hsing University, Taichung 402, Taiwan

## Abstract

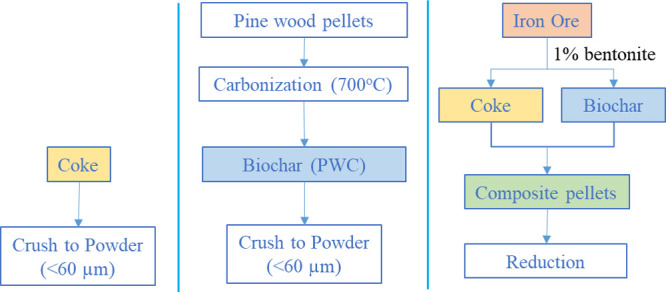

This study investigated the optimal proportion of biochar derived from pinewood pellets (PW) and coke as reducing agents for the carbothermal reduction of iron ore at high temperatures. Thermogravimetric analysis, elemental analysis, X-ray fluorescence, and scanning electron microscopy were used to characterize the raw materials. To determine the effect of biochar proportion on reduction efficiency, presented as metallization, metallized pellets were subjected to chemical analysis, including total iron (T.Fe) analysis, metallic iron (M.Fe) analysis, and residual Fe_2_O_3_ and FeO analysis. The results indicated that the addition of biochar derived from PW, with coke as a reducing agent, considerably increased the efficiency of carbothermal reduction. Optimal reduction conditions were established at a reduction temperature of 1300 °C and a holding time of 20 min, with 20% coke and 80% pinewood char. In summary, biochar derived from PW can be used as an alternative to coke as a reducing agent in the iron reduction process. In addition, biomass can be used as a reducing agent to mitigate carbon consumption by reducing the amount of coke required in iron production.

## 1 Introduction

Although the iron and steel industry plays a key role in global growth and the global economy, it also substantially contributes to energy consumption and carbon emissions. By 2050, the demand for steel is expected to sharply increase, with steelmaking representing one of the most energy- and carbon-consuming sectors.^1^ Although reducing energy consumption and gas emissions is regarded as a top priority, in recent years, achieving such reduction targets has been offset by the increasing scale of production, which, in turn, has resulted in an increase in CO_2_ emissions worldwide. Therefore, as an alternative to fossil fuels, renewable biomass has been proposed as a viable source of heat and a reducing agent aimed at reducing CO_2_ emissions.^2,3^ Thus, achieving synergy between biomass-based and steelmaking sectors is crucial for achieving enhanced performance, efficiency, and sustainability. Due to its reliance on nonrenewable energy sources, such as coal, for iron reduction, the iron and steel manufacturing industry is regarded as a major contributor to global pollution. Therefore, given the increasing environmental concerns and the demand for eco-friendly alternatives, exploring alternative carbon sources for iron reduction has become a top priority. To this end, biomass has emerged as a viable alternative to fossil fuels in that it provides a renewable and sustainable source of energy.^4^ Derived from biodegradable waste and residues produced by multiple industries, biomass can be used to generate bioenergy and to manufacture a range of biobased products.^5^

The iron and steel industry is a significant contributor to environmental challenges, particularly in terms of high carbon emissions. A potential solution to mitigate these issues involves the utilization of biomass, offering a renewable and lower carbon alternative to conventional fossil fuels. This shift has the potential to diminish the industry’s overall carbon footprint. Additionally, incorporating biomass can diversify energy sources and enhance the quality of iron production.^6^ Notably, pinewood (PW) derived from the forest industrial sector emerges as a substantial biomass waste, representing one of the most abundant tree varieties globally. The byproduct of pinewood, in the form of sawdust pellets, stands out as a promising biofuel feedstock. Upon conversion to biochar, these pellets become a rich source of high-content carbon, further contributing to the efficacy of our sustainable approach and promoting eco-friendly practices. In this study, PW was used to produce biochar through a thermochemical process known as slow pyrolysis or carbonization. It is a thermal pretreatment technique that enhances the properties of biomass to increase its reduction efficiency^7^ which involves the use of heat to transform biomass into energy and chemical products. This process is applicable to produce solids (biochar), liquids (biocrude), and gases (syngas) without pollution.^8^ Pinewood char (PWC), appears to be particularly promising for iron reduction applications because of its high carbon content and unique properties, including high porosity and large surface area, both of which facilitate the entrance of gases into pellet pores. Furthermore, biomass provides a promising foundation for both metallurgy and energy production, with the overarching goal of minimizing CO_2_ emissions. To reduce fossil fuel consumption and greenhouse gas emissions, environmental concerns pertaining to the iron and steel manufacturing industry must be addressed, and the optimal proportion of biomass suitable for use as a carbon source in iron reduction must be identified.^1^ Accordingly, studies should focus on increasing the efficiency of the current ironmaking base biochar and developing new technologies to understand the reduction characteristics of biochar. Exploring these alternative carbon sources and increasing their efficiency could promote the employment of more sustainable and eco-friendly approaches for iron reduction. During iron reduction, a carbon-reducing agent, namely, coke or biochar, is mixed with iron ore powder in appropriate proportions to form composite pellets. In carbothermal reduction, carbon is used at high temperatures, and fixed carbon serves as the primary reducing agent.^9^ According to Franklin’s research,^10,11^ coke derived from fossil fuels, such as coal, can undergo graphitization when heated above approximately 2200 °C. In contrast, biochar produced from biomass remains resistant to transformation into crystalline graphite, even when subjected to temperatures as high as 3000 °C.

Therefore, the objective of this study is to develop a novel and clean ironmaking technology incorporating PWC as a reducing agent in iron ore reduction. The investigation focused on examining the properties of biochar used under multiple reduction conditions including the proportion ratio between coke and biochar. In addition, the reduction behavior of the composite pellets was also studied. The results of this study bear substantial implications for the iron and steel manufacturing industries, particularly in the context of mitigating carbon emissions and advocating for a more sustainable and eco-friendly method in the process of iron reduction.

## 2 Materials and Methods

### 2.1 Feedstock

**Figure 1:**
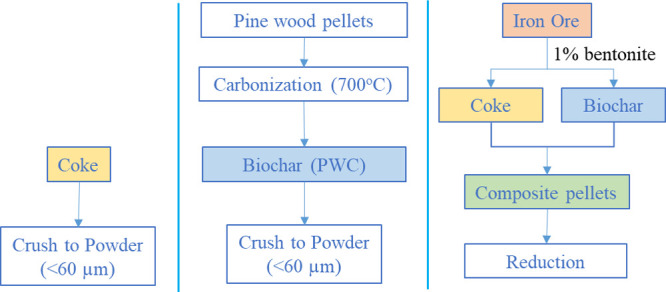
Flowchart of the experimental procedure.

**Figure 2:**
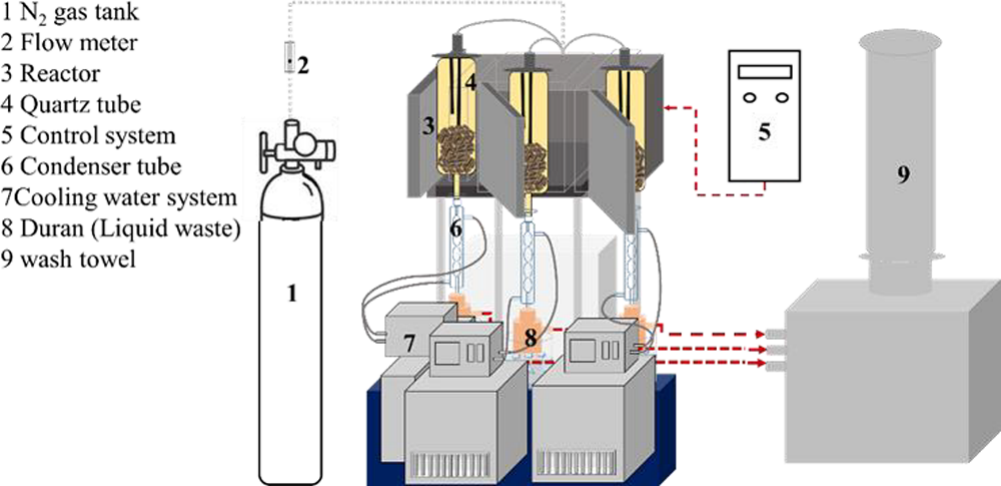
Schematic of the carbonization process.

In this study, iron ore powder (synthesis-grade Fe_2_O_3_) with a particle size of less than 60 μm was used as the raw material for the production of reduction process and to assist in the formation of composite pellets, where 1% bentonite was added as a binder. The chemical compositions of the iron ore and bentonite are shown in Table 1, revealing the Fe_2_O_3_ content of 96%, total iron (*T.Fe*) content of 70.83%, and low concentrations of Al_2_O_3_, SiO_2_, K_2_O, CaO, and MnO. Coke (China Steel Corporation, Taichung, Taiwan) and commercial PW were used as reducing agents. Table 2 presents the proximate and ultimate analysis results related to PW and coke. Figure 1 shows a flowchart of the experimental procedure. To produce pinewood char (PWC), PW was subjected to carbonization in a batch multisectional, temperature-controlled reactor with a volume of 2.8 L under a nitrogen atmosphere with a flow rate of 1.5 L/min (Figure 2). The carbonization temperature was set to 700 °C and the holding time for 1 h. After the carbonization process, the biochar underwent grinding and sieving, resulting in particle sizes of 40–60 and <60 μm. These specific sizes were chosen for both analysis purposes and utilization in the experimental procedures.

**Table 1:** Chemical Compositions of Iron Ore and Bentonite (Mass%)

ion ore (mass%)							
constituents	total Fe	Fe_2_O_3_	Al_2_O_3_	SiO_2_	K_2_O	CaO	MnO
	70.83	96.00	0.05	2.08	0.05	0.01	0.05

bentonite (mass%)						
constituents	Na_2_O	Al_2_O_3_	SiO_2_	K_2_O	CaO	MgO
	0.84	13.86	71.10	0.50	1.50	0.81

**Table 2:** Proximate and Ultimate Analyses of Feedstock^a^

feedstock	proximate analysis (wt %, dry)				ultimate analysis (wt % daf)				
	ash	VM	FC	HHV (MJ/kg)	C	H	O	N	S
coke	12.47	12.70	74.83	28.95	95.60	0.16	2.40	1.07	0.77
PWC	1.32	13.08	85.60	31.57	91.52	1.37	6.61	0.50	0

a VM, volatile matter; FC, fixed carbon; HHV, higher heating value.

### 2.2 Composite Pellet Preparation and Experimental Procedure

**Figure 3:**
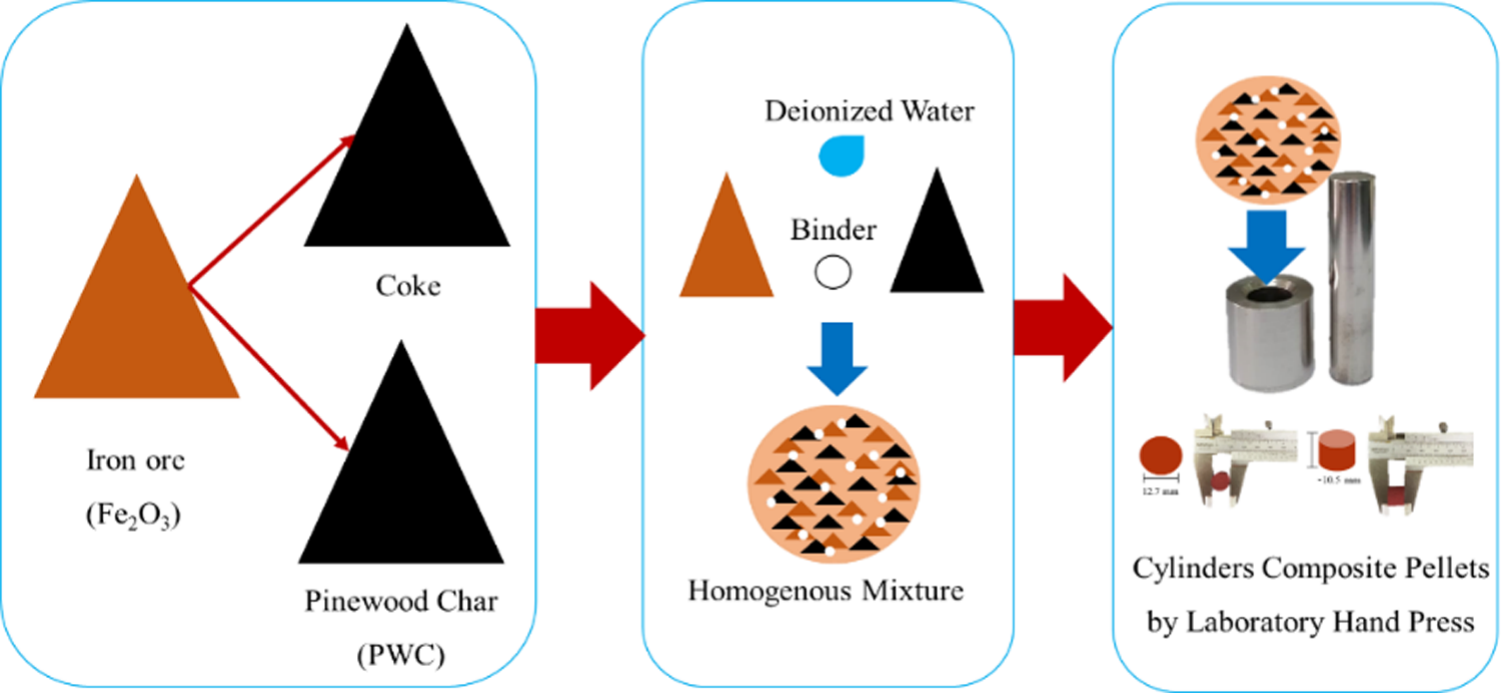
Composite pellet creation process.

**Figure 4:**
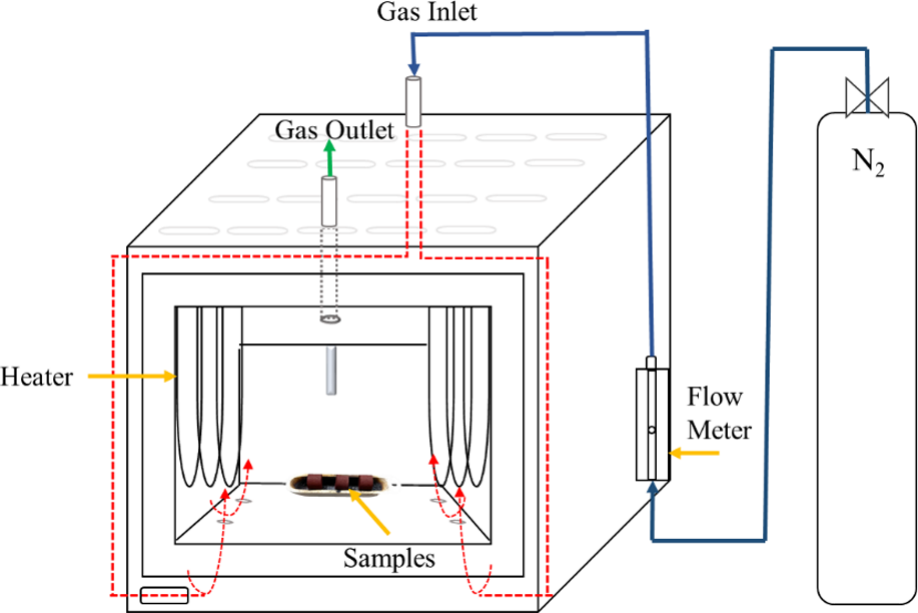
Carbothermal reduction equipment (electric muffle furnace with a maximum working temperature of 1300 °C).

For the preparation of composite pellets for the reduction process, 20 g of iron ore powder, a combination of the reducing agent (coke and biochar), and 1% bentonite were used, as illustrated in Figure 3. The creation of composite pellets involved using a laboratory hand press to shape cylindrical pellets with a diameter of 12.7 mm, height of 10 mm, and weight ranging from 2.0 to 2.2 g. Subsequently, these composite pellets underwent a drying process in a hot air oven at 105 °C for 12 h before being subjected to carbothermal reduction within an electric muffle furnace, as depicted in Figure 4. In each experimental trial, three composite pellets were employed. The reduction process took place at temperatures of 1200 and 1300 °C for durations of 20 and 25 min, respectively, with a continuous flow of nitrogen gas into the furnace at a rate of 2 L/min. Following the reduction process, the samples were allowed to cool within the furnace before undergoing analysis. The specific reduction conditions and the proportion ratio of composite pellets utilized in each experiment are detailed in Table 3.

**Table 3:** Reduction Conditions for Carbothermal Reduction of Composite Pellets^a^

sample name	reducing agent	ratio (%)	iron ore (g)	loading (g)	reduction temp. (°C)	reduction time (min)
pellet A	coke	100	20	2–2.2	1200	20
pellet B	PWC	100				
pellet C	coke:PWC	80:20				
pellet D	coke:PWC	50:50			1300	25
pellet E	coke:PWC	20:80				

a PWC, pinewood char.

### 2.3 Analysis

#### 2.3.1 Characterization

Following carbonization, a comprehensive set of analyses was conducted to explore the characteristics of carbonized biomass. Proximate analysis, ultimate analysis (elemental composition), higher heating value (measured with a Parr 6300 calorimeter), elemental composition assessment using X-ray fluorescence, and surface morphology examination through scanning electron microscopy were all performed. Additionally, thermogravimetric analysis (TGA), carried out with a PerkinElmer STA6000 instrument, provided insights into the behavior of biomass and its constituents during carbonization. Furthermore, the specific surface area of the feedstock and the physical adsorption of gas molecules on a solid surface were evaluated through porous analysis using the Brunauer–Emmett–Teller (BET) method.

#### 2.3.2 Metallization

The metallized pellets were subjected to a chemical analysis. The pellet was ground into a powder. Then, it was used to measure the concentrations of metal iron (M.Fe), total iron (T.Fe), and residual FeO by the potassium dichromate titration process. The prereduction indicator methylene blue and the reduction agent stannous chloride were used to construct a method for determining T.Fe in composite pellets.^12^ For M.Fe analysis, an iron trichloride solution was used to dissolve the substance. Before going into the solution, metallic iron in the sample was oxidized to ferrous chloride, and iron(II) remained in the sediment before being filtered and separated. Then, the solution was titrated with potassium dichromate standard solution, with sodium diphenylamine sulfonate as the indicator.^13^ The experiment was performed with three repetitions for one sample. Metallization (%) was calculated as in eq 1.
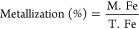
1where M.Fe represents metallic iron, and T*.*Fe represents total iron in the sample.

## 3 Results and Discussion

### 3.1 Characterization of Raw Materials

The characterization of raw materials, such as pinewood char and coke, plays a major role in understanding their suitability as reducing agents. In ironmaking, wood biomass can be used instead of coal and coke to achieve improved energy security and reduced greenhouse gas emissions.^1^ Wood biomass can also be used as a sustainable and renewable source of energy. In the present study, the proximate analysis results revealed that the fixed carbon content of PWC (85.60%) was considerably higher than that of coke (74.83%), indicating that PWC can be used as an efficient reducing agent. Therefore, understanding the properties of these raw materials is crucial to determining their effectiveness as reducing agents. Compared with coke, the higher volatile matter content of PWC (13.08% vs 12.70%) and the lower ash content of PWC (1.32 vs 12.47%) suggest that PWC can offer great benefits as a reducing agent in ironmaking. These findings have major implications for the development of a sustainable and eco-friendly ironmaking technique that involves the use of biomass as an alternative carbon source.^14^

PWC exhibited a higher heating value (HHV) of 31.57 MJ/kg, which was higher than that of coke (28.95 MJ/kg; Table 2). As oxygen in the feedstock is consumed during the carbonization process, there was a decrease in C–O bonds in woody biomass. The rise of HHV is caused by an increased carbon content due to a higher C–C bond energy.^15^

**Figure 5:**
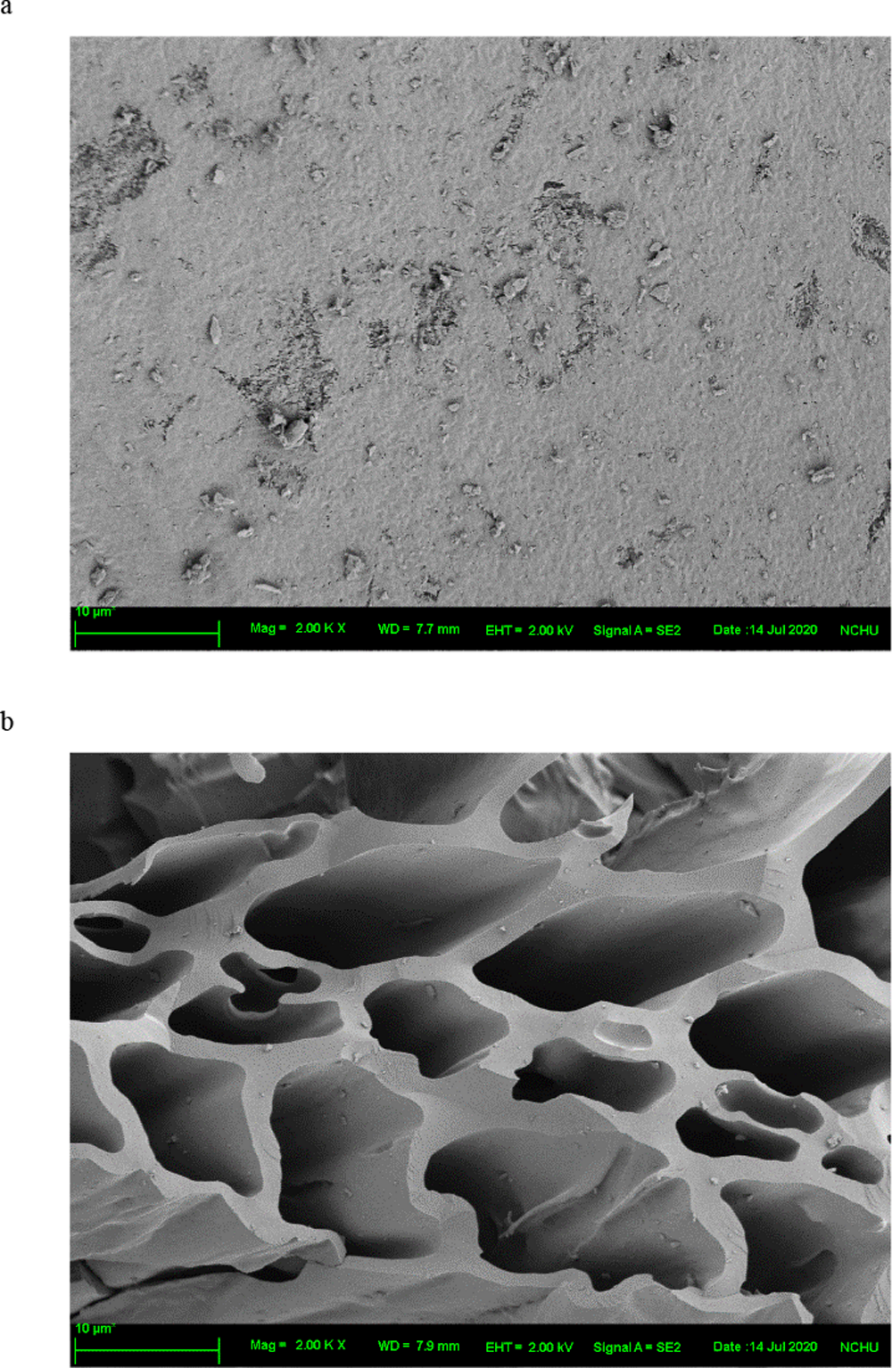
Scanning electron microscopy images of reducing agents: (A) coke and (B) pinewood char (700 °C).

As shown in Figure 5, morphological analysis revealed that coke had a smooth surface with closely linked particles, whereas PWC had a large porous structure with clear pipelines. The porosity of PWC enhanced the degree of reactivity during the reduction of iron ore, resulting in strong solid–gas interaction and improved reducing gas at high temperatures.^16^ Biochar with a high porosity and surface area typically has the ability to adsorb. According to BET analysis (Table 4), a specific surface area (SSA) of PWC was 77.133 m^2^/g, which was substantially higher than that of coke (9.225 m^2^/g). PWC has a highly porous structure, which is responsible for the effective reduction of iron ore. During pyrolysis, volatile matter is released, leading to the formation of pores, which in turn facilitates the rapid diffusion and efficient use of reducing gases in reduction.^15^ At high temperatures, biochar carbonization generates reducing gases, which further increases the efficiency of reduction. Moreover, PWC had a carbon content of approximately 91.52%, which is considered high for a carbon material and comparable to that of coke (95.60%). Compared with coke, PWC contains less sulfur and thus may increase the efficiency of reduction^18^ and makes it an environmentally friendly option. Overall, the characteristics of PWC suggest that it has great potential as a reducing agent in iron ore reduction, especially given its higher sustainability and eco-friendliness compared with coke. These results imply that using biomass, such as PWC, to reduce iron ore not only improves reduction efficiency but also contributes to preserving the environment.

**Table 4:** BET Analysis of Feedstock^a^

feedstock	carbonization temperature(°C/min)	SSA(m^2^/g)	TPV(cm^3^/g)	MPD(nm)
coke		9.225	0.013	5.830
PW	raw	1.725	0.008	19.160
	700	77.133	0.041	2.133

a SSA = specific surface area (m^2^/g), TPV = total pore volume (cm^3^/g), MPD = mean pore diameter (nm).

### 3.2 Effect of Biochar Addition on Carbothermal Reduction

**Figure 6:**
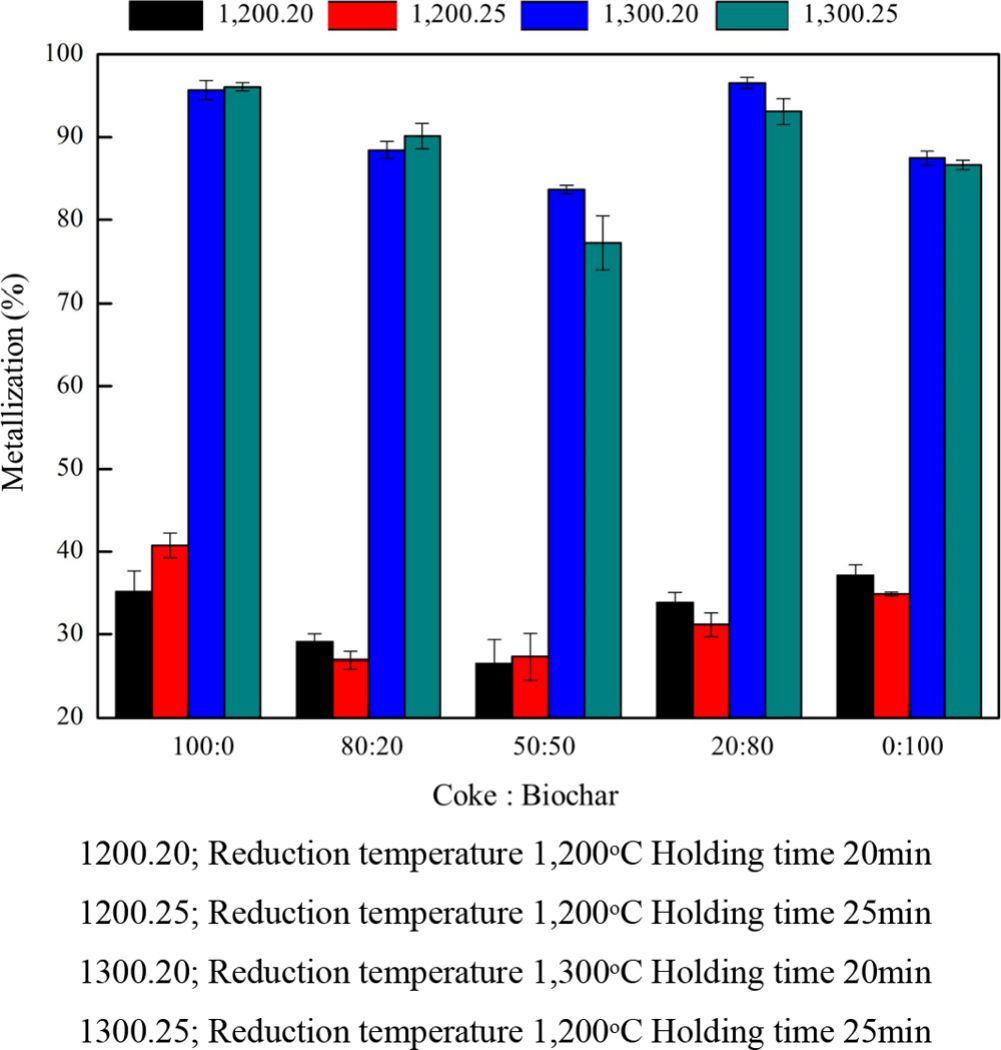
Effect of different reduction temperatures and holding times on the coke and biochar composite pellets to metallization %.

Biochar is a carbonaceous solid produced by the pyrolysis of biomass. It has the potential for use as a feedstock in the ironmaking industry. By replacing coke, a traditional reducing agent, with biochar, the ironmaking industry can reduce its environmental footprint, including its greenhouse gas emissions, air pollution, and water consumption.^19^ Biochar can also be used in the ironmaking industry to generate revenue for biomass power plants and transform their waste into valuable resources. In this study, a reduction experiment was conducted with multiple ratios of PWC to coke (i.e., 100:0, 80:20, 50:50, 20:80, and 0:100). Figure 6 illustrates the impact of different reduction temperatures and holding times on the metallization percentage in coke and biochar composite pellets. The results suggest that incorporating biochar contributes to improved metallization by facilitating more effective reduction processes, as depicted in the graph. Conversely, the results indicate that using 100% biomass can serve as a complete substitute for coke as a reducing agent, as evidenced by the metallization outcomes. The metallization substantially increased with the higher proportion of biochar, particularly when adding 80% biochar to the composite pellet at a reduction temperature of 1300 °C and a holding time of 20 min. However, although experiments incorporating biochar consistently yield excellent metallization results compared to those using only coke in this study, the differences in metallization are not significantly pronounced. However, when only coke was used as a reducing agent, metallization was lower than that when biochar was added. At a reduction temperature of 1200 °C, metallization decreased for all the aforementioned ratios. These findings suggest that a higher temperature and a longer holding time are necessary for achieving higher metallization with biochar as a reducing agent. It is noted that applying some biochar for iron ore reduction can also successfully increase the iron ore capacity for reduction. This allows iron and steel companies to encourage green development.

### 3.3 Reduction Behavior on Composite Pellets

**Figure 7:**
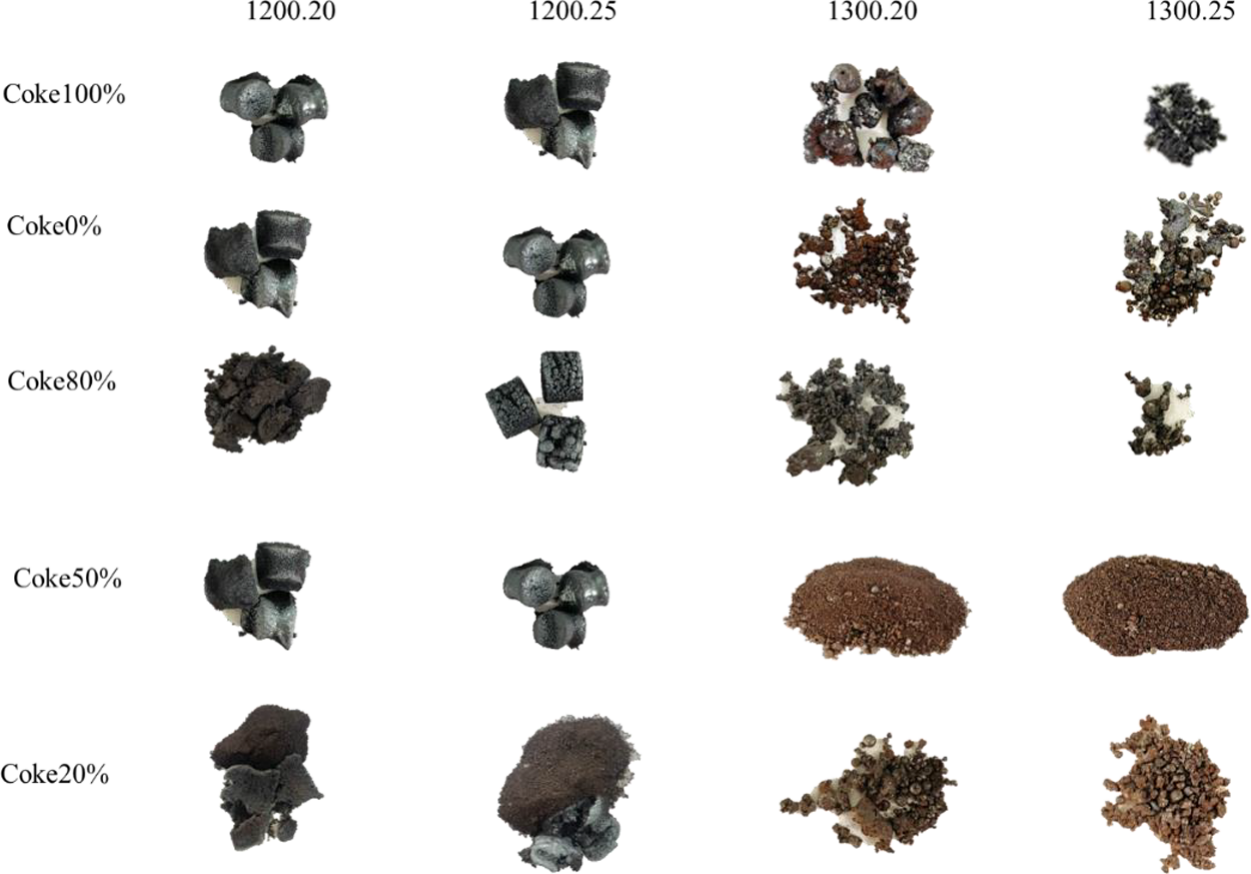
Appearance of composite pellets after reduction.

**Figure 8:**
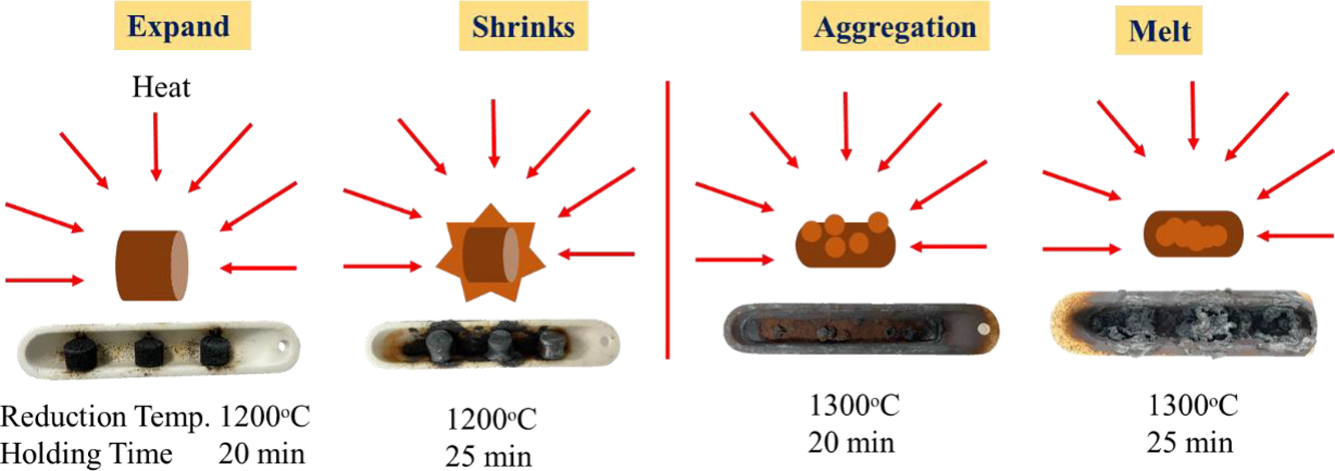
Schematic of composite pellets after reduction.

Following reduction with multiple proportions of carbon-reducing agents, changes in the structure of composite pellets were investigated; the results are presented in Figure 7. As the temperature and reduction time were increased, the appearance of the composite pellets was noticeably changed. For instance, at a reduction temperature of 1200 °C, slight changes were observed in the composite pellets, such as lining expansion after 20 min and aggregation after 25 min. At a reduction temperature of 1300 °C, a substantial increase in metallization was observed, with the most effective reduction identified with composite pellets containing 80% biochar and 20% coke. The composite pellet reduction in a low oxidizing environment was facilitated by the emission of volatiles, which led to a more porous structure. On the one hand, mass transfer was improved throughout the volatile reduction phase, and the release of volatility encouraged the transition from a solid–solid to a gas–solid reaction. Figure 8 presents a schematic of the composite pellets after reduction, indicating that their shrinkage can be attributed to the loss of carbon and oxygen during the reduction process.

In reduced pellets, the reducibility of iron oxide is affected by multiple factors, such as the carbonization temperature, reduction temperature, heating profile, and coal content. At a low maximum carbonization temperature, the highest reducibility is observed in pellets that contain semi-Newcastle blend coal char.^20^ During the reduction of carbon-containing iron ore pellets, direct and indirect reduction (endothermic) occur simultaneously. Increasing the temperature facilitates both indirect reduction through reaction kinetics and direct reduction through reaction thermodynamics.^21^ In addition, such an increase enables the total escape and pyrolysis of volatile matter in biomass char, resulting in an increase in the degree of reduction. In the present study, the effects of temperature and holding time were investigated at 1200 and 1300 °C and 20 and 25 min, respectively.

**Figure 9:**
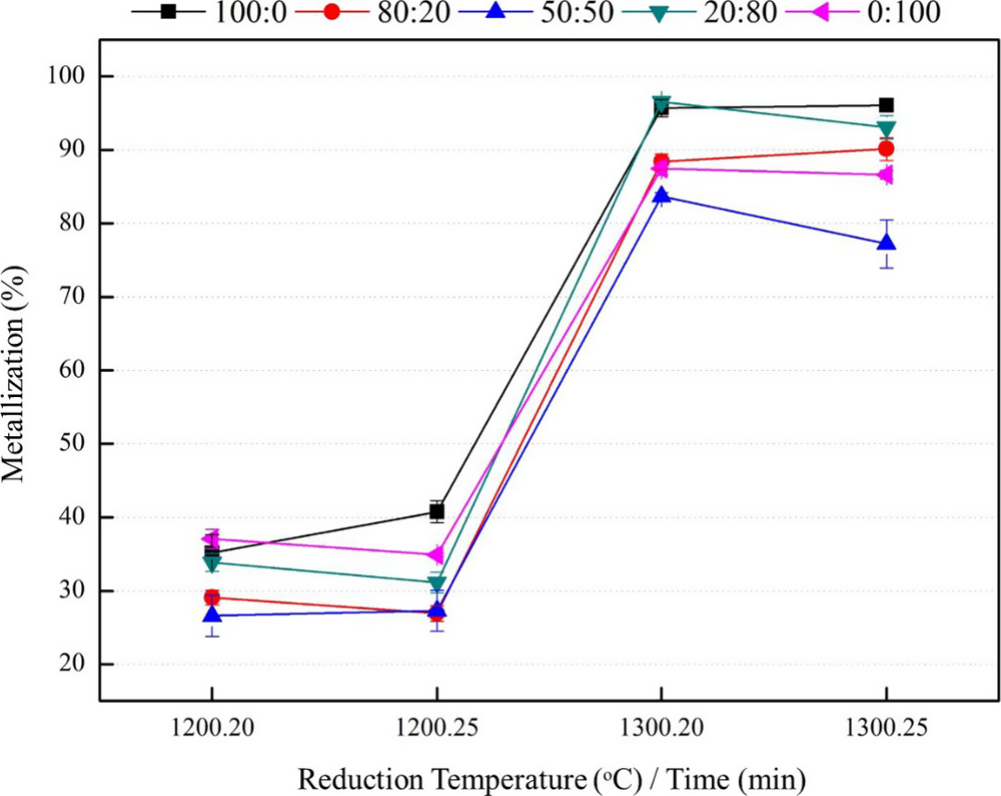
Effect of various coke and biochar proportions with different reduction temperatures and holding times on the metallization percentage.

The effect of various coke and biochar proportions (100:0, 80:20, 50:50, 20:80, and 0:100) at different reduction temperatures and holding times on the metallization percentage is shown in Figure 9. The graph indicates that metallization was less than 50% for composite pellets roasted at 1200 °C but as high as 86.63–96.08% for those roasted at 1300 °C. The effect of holding time on metallization was investigated with biochar as the reducing agent. The results indicated no substantial difference in metallization between holding times of 20 and 25 min, indicating that the metallization process required a holding time of at least 20 min. This finding was consistent with that of Yuan et al.,^17^ who reported that the degree of pellet metallization exceeded 85% with 20 min of reduction but then rapidly decreased when reduction was resumed for 40 and 60 min.

### 3.4 Residual Fe_2_O_3_ and FeO Analysis

**Figure 10:**
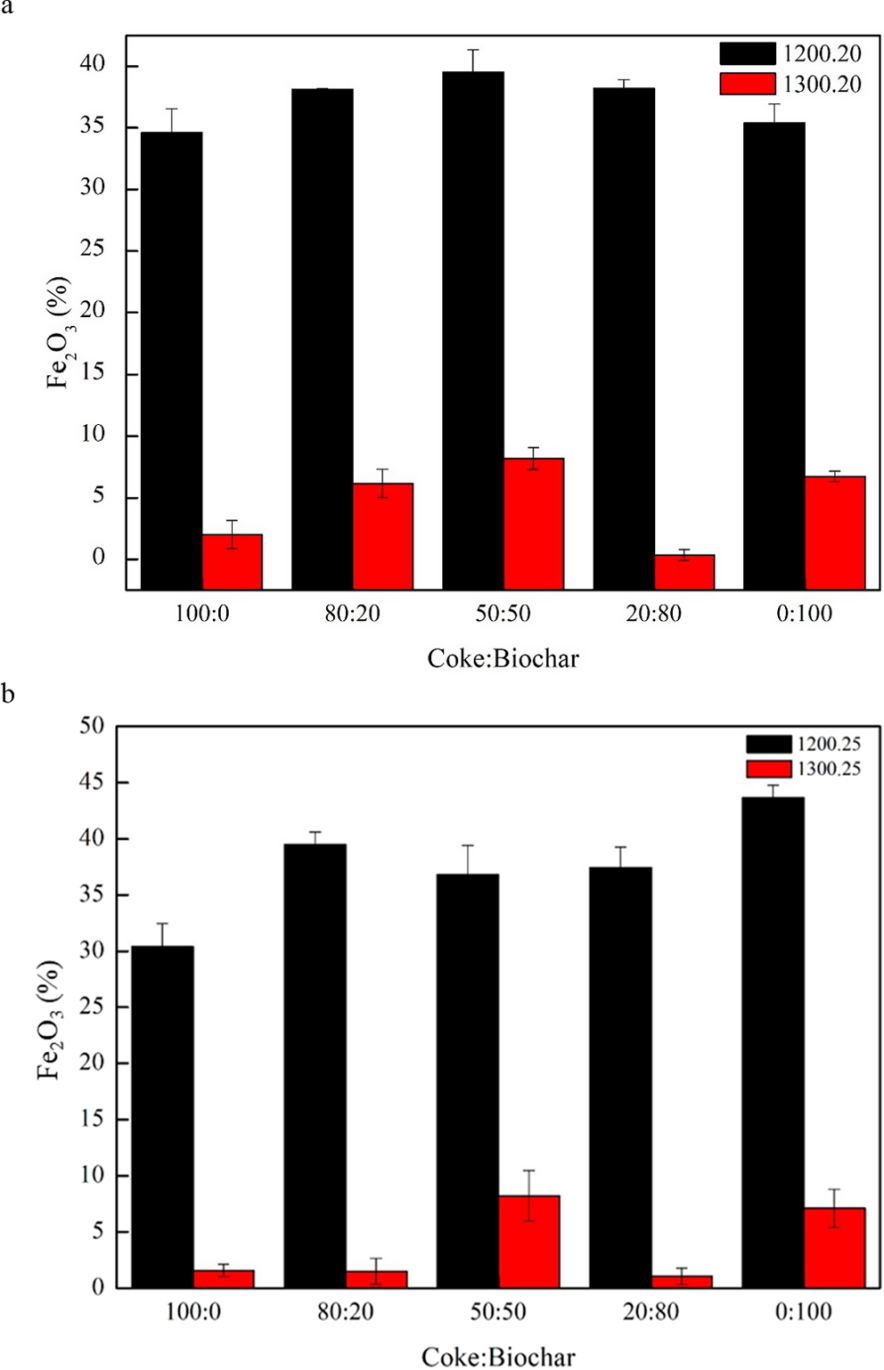
Analysis of residual Fe_2_O_3._.

**Figure 11:**
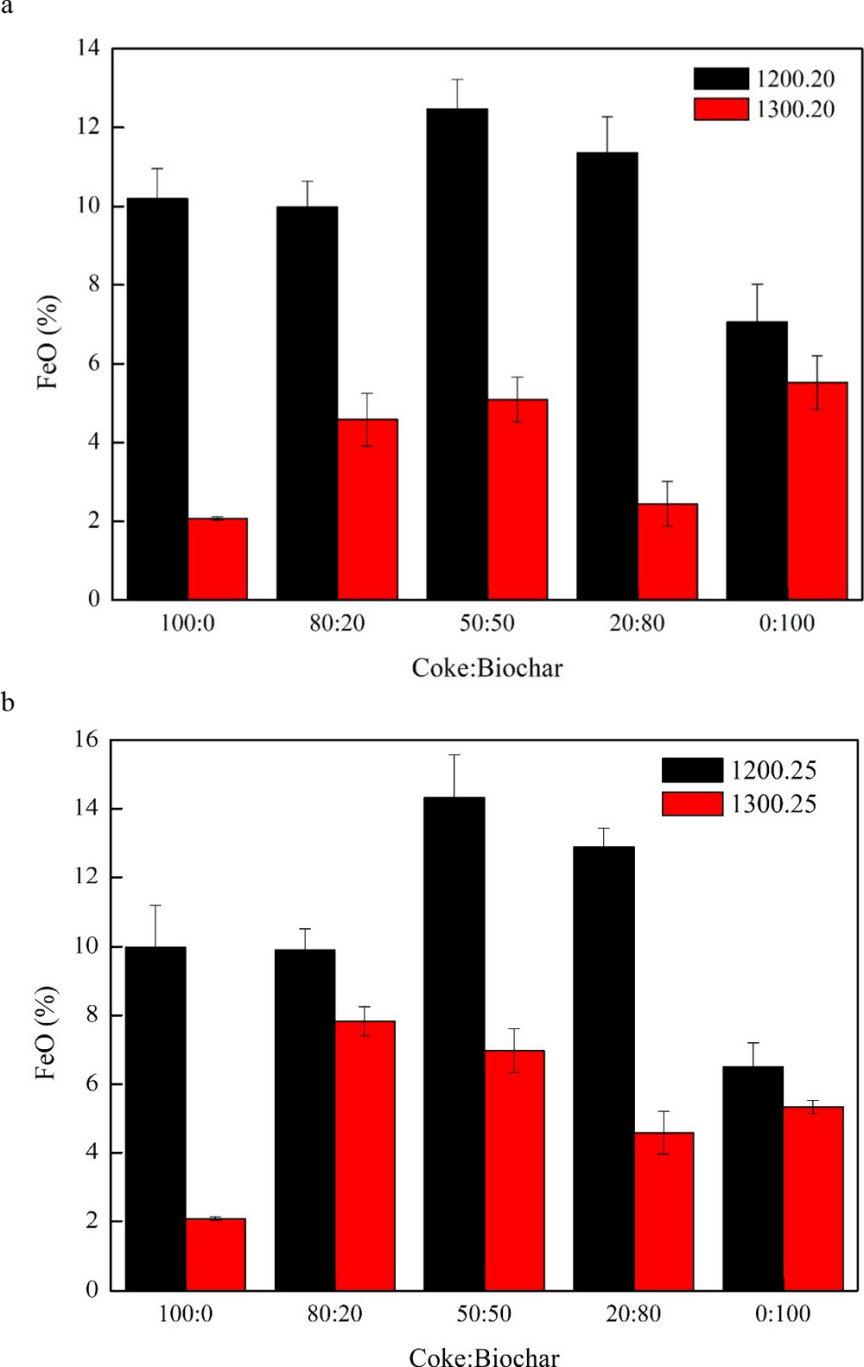
Analysis of residual FeO.

Reducing composite pellets containing coke and biochar offers an alternative method for producing iron with only a minor environmental footprint.^22^ In the present study, the potential of biochar as a reducing agent was explored, and its effectiveness was compared with that of coke. The results indicated that biochar can be used as a reducing agent thanks to the reduction efficiency that increases with temperature. This finding suggests that biochar has the potential to reduce the amount of coke required for iron production and thus can mitigate the overall cost and environmental concerns.^23^ The presented study of Fe_2_O_3_ and FeO residue analysis was conducted on composite pellets containing varying proportions of coke and biochar.^24^Figures 10 and 11 present a graph of Fe_2_O_3_ and FeO residue percentages. At a reduction temperature of 1200 °C, the proportion of the Fe_2_O_3_ residue exceeded 30%, whereas at a reduction temperature of 1300 °C, it fell below 10%. When the reduction temperature of 1300 °C was held for 20 min, metallization improved, leading to the production of metallic iron (FeO → Fe). Metallic iron is preferentially produced at higher temperatures above 1200 °C. According to Han et al.,^25^ at temperatures below 1000 °C, iron oxides undergo the following reduction reaction: Fe_2_O_3_ → Fe_3_O_4_ → FeO.

In addition to temperature control, the duration of reduction plays a crucial role in determining the quality of the produced iron. Specifically, maintaining pellets at high temperatures for extended durations improves metallization and increases the yield of metallic iron,^26^ thereby improving the quality of the produced iron and increasing the efficiency of the ironmaking process. Furthermore, the use of biochar as a reducing agent in ironmaking has several environmental benefits. For instance, when biochar is used as a reducing agent, waste materials can be transformed into useful products, and greenhouse gas emissions are reduced. Accordingly, future research should investigate the potential of biochar as a reducing agent in ironmaking and examine the effects of multiple parameters—including particle size, holding time, and reduction temperature—on the reduction and the quality of the produced iron. Further studies should also focus on the use of biochar as a reducing agent in other high-temperature reduction processes with a view to developing more sustainable and eco-friendly industrial processes.

## 4 Conclusions

In this study, different proportions of carbonaceous materials were used for iron reduction with biochar derived from pinewood pellets through a pyrolysis process. While using agricultural waste, like pinewood, for biochar in ironmaking has potential benefits, it is crucial to consider the specific requirements of the ironmaking process such as the biochar characteristics. These biochar offer a high content of carbon source of about 91.52 wt % and zero sulfur dioxide, contributing to improved process efficiency and lowered greenhouse gas emission compared to traditional carbon sources. Moreover, the porosity and surface area of biochar can enhance reactivity, making it a potential sustainable alternative in ironmaking, ensured by BET and morphology analyses, which can serve as a reducing agent in the iron reduction process.

The addition of a biochar substitute for coke as a reducing agent for the reduction process can improve the iron reduction efficiency. Every treatment that added biochar had a good result. However, the treatment with 80% biochar showed the best result compared to other treatments and was even better than 100% coke. At a reduction temperature of 1300 °C and a holding time of 20 min, metallization increased by more than 90%. The observed outcomes can be attributed to the meticulous consideration of the appropriate proportion and conditions, the elevated carbon content, and volatile matter content of biochar. Additionally, the larger specific surface area (SSA) of biochar played a crucial role by facilitating easier gas diffusion into the pellet pores. This unique combination of factors rendered biochar exceptionally efficient in the process of converting iron oxide to metallic iron.

In summary, the use of certain biochar for iron reduction can effectively improve the reduction capacity of iron ore and can also contribute to the green development of ironmaking. While both biochar and coke can be used as reducing agents in ironmaking processes, biochar has certain limitations. It generally has lower reactivity and strength compared with coke, which may impact its efficiency in traditional blast furnace operations. Research is ongoing to optimize biochar’s properties for ironmaking, but currently, coke remains the primary choice due to its well-established performance and availability. Additionally, the use of biochar as a reducing agent can save carbon consumption in the ironmaking process and promote sustainability and environmental friendliness.
